# *Plasmodium vivax* merozoite-specific thrombospondin-related anonymous protein (PvMTRAP) interacts with human CD36, suggesting a novel ligand–receptor interaction for reticulocyte invasion

**DOI:** 10.1186/s13071-023-06031-5

**Published:** 2023-11-19

**Authors:** Thau Sy Nguyen, Ji-Hoon Park, Tuyet-Kha Nguyen, Truong Van Nguyen, Seong-Kyun Lee, Sung-Hun Na, Jin-Hee Han, Won-Sun Park, Wanjoo Chun, Feng Lu, Eun-Taek Han

**Affiliations:** 1 Department of Medical Environmental Biology and Tropical Medicine, School of Medicine, Kangwon National University, Chuncheon, Gangwon-Do 24341 Republic of Korea; 2 Host-Parasite Research Laboratory, Institut Pasteur Korea, Seongnam-Si, 13488 Republic of Korea; 3 Department of Obstetrics and Gynecology, School of Medicine, Kangwon National University, Chuncheon, Gangwon-Do 24341 Republic of Korea; 4 Department of Physiology, School of Medicine, Kangwon National University, Chuncheon, Gangwon-Do 24341 Republic of Korea; 5 Department of Pharmacology, School of Medicine, Kangwon National University, Chuncheon, Gangwon-Do 24341 Republic of Korea; 6 Department of Pathogen Biology and Immunology, School of Medicine, Yangzhou University, Yangzhou, China

**Keywords:** *Plasmodium vivax*, Ligand, Receptor, PvMTRAP, CD36, Interaction

## Abstract

**Background:**

The *Plasmodium vivax* merozoite restrictively invades immature erythrocytes, suggesting that its ligand(s) might interact with corresponding receptor(s) that are selectively abundant on reticulocytes to complete the invasion. Finding the ligand‒receptor interaction involved in *P. vivax* invasion is critical to vivax malaria management; nevertheless, it remains to be unraveled.

**Methods:**

A library of reticulocyte receptors and *P. vivax* ligands were expressed by a HEK293E mammalian cell expression system and were then used to screen the interaction using enzyme-linked immunosorbent assay (ELISA). A flow cytometry-based erythrocyte binding assay and bio-layer interferometry experiment were further utilized to cellularly and quantitatively identify the ligand‒receptor interaction, respectively.

**Results:**

*Plasmodium vivax* merozoite-specific thrombospondin-related anonymous protein (PvMTRAP) was found to interact with human CD36 using systematic screening. This interaction was specific at a molecular level from in vitro analysis and comparable to that of *P. vivax* Duffy binding protein (PvDBP) and Duffy antigen receptor for chemokines (DARC) (*K*_D_: 37.0 ± 1.4 nM and 7.7 ± 0.5 nM, respectively). Flow cytometry indicated that PvMTRAP preferentially binds to reticulocytes, on which CD36 is selectively present.

**Conclusions:**

Human CD36 is selectively abundant on reticulocytes and is able to interact specifically with PvMTRAP, suggesting that it may function as a ligand and receptor during the invasion of reticulocytes by *P. vivax*.

**Graphical Abstract:**

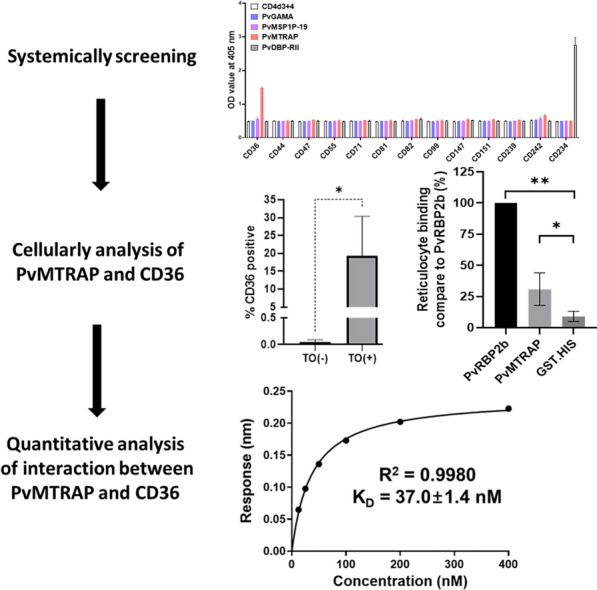

**Supplementary Information:**

The online version contains supplementary material available at 10.1186/s13071-023-06031-5.

## Background

Malaria currently remains a major global public health burden, as nearly half of the population is at risk of infection, with an estimated 247 million and 619,000 people developing clinical diseases and dying in 2021, respectively [1]. *Plasmodium* spp., causative agents of malaria, can maintain cycles between the invasion of and egress from erythrocytes, resulting in the asexual blood stage, which accounts for most of the clinical manifestation of malaria. Once released, merozoites rapidly invade erythrocytes through a series of mechanical steps that fall into three main phases: the first, in which the merozoite initially interacts with and deforms the erythrocyte membrane; the second, which involves apical interaction and invasion; and the third, which involves resealing of the erythrocyte cytoskeleton and restoration of the invaded red blood cell [2, 3].

Among *Plasmodium* species causing malaria in humans, *Plasmodium vivax* is the most widely geographically spread [4]. Additionally, hypnozoites produced by *P. vivax* can lead to recurring infections, and a lack of sufficient treatment for this dormant form has been seen as a vital barrier to the eradication of malaria [5, 6]. Vaccination targeting the erythrocytic stage appears to be a critical preventative method, leading to demand for elucidation of the ligand‒receptor interactions that are used by *P. vivax* during the reticulocyte invasion process [7, 8]. *Plasmodium vivax* Duffy binding protein (PvDBP) and the red blood cell Duffy antigen receptor for chemokines (DARC) are the most well-known and well-studied ligand–receptor pair [9, 10]. However, an accumulation of *P. vivax* infection cases among individuals lacking DARC suggests the existence of an alternative ligand and receptor for invasion [11, 12]. Moreover, the distribution of DARC on reticulocytes is not considerably different from that on mature erythrocytes; hence, this possibility fails to explain the restriction to reticulocytes of *P. vivax* [13]. Recently, transferrin receptor 1 (TfR1 or CD71), which is an abundant reticulocyte protein, was explored as the receptor of *P. vivax* reticulocyte-binding protein 2b (PvRBP2b) [14]. Nevertheless, CD71 is sensitive to trypsin treatment, inconsistent with the trypsin resistance in *P. vivax* merozoite invasion [15, 16], and antibodies against CD71 cannot abolish merozoite invasion [14]. Furthermore, a study has demonstrated that CD98, a reticulocyte-specific protein resistant to trypsin, binds to PvRBP2a during merozoite invasion. Nonetheless, PvRBP2a also binds to normocytes, and the binding to reticulocytes is inhibited insufficiently by antibodies against CD98 compared to anti-PvRBP2a antibodies [17]. It is widely accepted that the parasite may utilize various protein ligands to interact with their corresponding receptors on erythrocytes for invasion; however, most ligand‒receptor interactions remain to be identified and/or characterized [18].

The thrombospondin-related anonymous protein (TRAP) family contains many proteins that share conserved structural and functional features related to adhesion [19]. In *Plasmodium* spp., TRAP participates in parasite motility through adhesion and formation of the actin–myosin motor and subsequent invasion into the host cell [20]. TRAP has been included in various multivalent subunit vaccines as a component, and some of these vaccines are under assessment in clinical trials [20]. Localized to merozoite and expressed in the middle-to-late stage of the erythrocytic cycle, merozoite-specific TRAP (MTRAP) shows major structural and functional similarities to TRAP and is assumed to be involved in merozoite invasion [21]. The MTRAP protein from *Plasmodium falciparum* has been found to interact with its receptor on erythrocytes, semaphorin-7A (CD108) [22], strengthening the evidence for the role of MTRAP in erythrocyte invasion by merozoites and in turn accelerating research on MTRAP in the contexts of other human malaria parasites that have been mostly mysterious thus far.

Human cluster of differentiation 36 (CD36) participates in malaria pathophysiology with either protective or pathological roles [23]. Erythrocytes are among various human cell types expressing CD36, and this receptor has been considered to be abundant on immature red blood cells [24, 25], which are host cells of *P. vivax*. Furthermore, the human thrombospondin 1 (TSP-1) protein was known to interact with human CD36 by using its thrombospondin repeat (TSR) domain [26], which is also included in the *P. vivax* MTRAP (PvMTRAP) sequence [27], suggesting that PvMTRAP and CD36 on reticulocytes may induce ligand‒receptor interactions during the invasion process.

To assess whether *P. vivax* utilizes MTRAP for any interaction with reticulocytes, we included PvMTRAP in a systematic screening for interaction with a library of reticulocyte receptors. A flow cytometry-based reticulocyte binding assay and bio-layer interferometry (BLI) experiment were then used to cellularly and quantitatively identify the ligand‒receptor interaction, respectively.

## Methods

### Umbilical cord blood sampling

Cord blood samples were acquired in 10-ml heparin tubes (BD Vacutainer^®^, Becton, Dickinson and Company, Franklin Lakes, NJ, USA). All relevant guidelines and regulations were followed, and all experimental protocols involving human samples were approved by the Kangwon National University Hospital Ethical committee (IRB No. KNUH-B-2021-06-034). Written informed consent was obtained from all subjects.

### Recombinant protein expression and purification

All recombinant proteins were produced using the mammalian human embryonic kidney 293E (HEK293E) cell expression system as described elsewhere [28]. For plasmid preparation, pTT3 and pTT5 containing an appropriate exogenous signal peptide with intended C-terminal tags were generated. A group of reticulocyte receptor proteins and parasite ligand proteins were incorporated with Fc- and His-tags, respectively. The full-length ectodomain was chosen for expression, except for *P. vivax* reticulocyte binding protein 2b (PvRBP2b, residues 169 to 813), *P. vivax* GPI-anchored micronemal antigen (PvGAMA, residues 408 to 589), PvDBP (region II, residues 194 to 521), *P. vivax* merozoite surface protein 1 paralog-19 (PvMSP1P-19, residues 1751 to 1834), and DARC (CD234, residues 1 to 63). The complementary DNA (cDNA) of the intended sequences was codon-optimized for expression in mammalian cells using GeneArt (https://www.thermofisher.com/order/geneartgenes/projectmgmt) and then chemically synthesized by Twist Bioscience (South San Francisco, CA, USA). HEK cells were maintained in suspension in Gibco FreeStyle™ F17 Expression Medium (Life Technologies Corp., Grand Island, NY, USA) at 37 °C under 70% humidity and 8% CO_2_ in an orbital shaking incubator at 120 revolutions per minute. Twenty-four hours before transfection, fresh F17 medium was used to seed cells to a final density of 6 to 8 × 10^5^ cells/ml. For each transfection, the expression plasmid was mixed with polyethyleneimine MAX transfection reagent (Polysciences, Warrington, PA, USA) before transfection into HEK cells. To produce biotinylated proteins, D-biotin (Sigma-Aldrich, St. Louis, MO, USA) and a plasmid encoding a secreted form of *Escherichia coli* biotin ligase (BirA) were added during transfection. After 5 days of incubation post-transfection, culture supernatants containing protein were collected for protein purification. His-tagged proteins were purified using Ni–NTA agarose (QIAGEN, Hilden, Germany) and a Poly-Prep^®^ chromatography column (Bio-Rad, Hercules, CA, USA). HiTrap^®^ Protein G HP (Sigma-Aldrich) was used for the purification of Fc-fusion proteins. The purified proteins were subjected to buffer exchange with HEPES-buffered saline (HBS) and concentrated using a 30-kDa Amicon^®^ Ultra-15 Centrifugal Filter (Sigma-Aldrich).

### Sodium dodecyl sulfate–polyacrylamide gel electrophoresis (SDS‒PAGE) and western blotting

His- and Fc-tagged recombinant proteins were separated by 13% SDS‒PAGE. When needed, a reducing agent was added to the sample to induce reducing conditions. The gels for SDS‒PAGE analysis were stained with Coomassie brilliant blue (Sigma-Aldrich), while others were used for protein blotting onto polyvinylidene fluoride (PVDF) membranes (MilliporeSigma, Burlington, MA, USA), after which the membranes were blocked with 5% skim milk for 1 h. The membranes containing His-tagged protein were then incubated with an anti-Pena-His antibody (1:2000) (LI-COR^®^ Biosciences, Lincoln, NE, USA) and then with a secondary IRDye^®^ goat anti-mouse (1:10,000) antibody (LI-COR^®^ Biosciences), while only goat anti-human antibodies (1:5000) were used for Fc-fusion proteins (LI-COR^®^ Biosciences). Data analysis was performed with an Odyssey infrared imaging system and software (LI-COR^®^ Biosciences).

### Protein‒protein interaction screening by enzyme-linked immunosorbent assay (ELISA)

The biotinylated proteins were normalized on a streptavidin-coated plate with mouse anti-rat CD4 monoclonal antibody (clone: OX68, Novus Biologicals™, Centennial, CO, USA). After normalization, 100 µl of each biotinylated protein was incubated for 1 h at room temperature (RT) and then washed with phosphate-buffered saline with 0.1% Tween 20 (PBS-T) and PBS. Then, 1 μg of Fc-fusion protein in PBS-T was added, and the mixture was incubated for 1 h with shaking. Following washing steps with PBS-T, the plate was allowed to react with a goat anti-human Fc-specific alkaline phosphatase antibody (Sigma-Aldrich, 1:5000 in PBS-T) for 1 h at RT. After washing with PBS-T, 100 µl of 1-Step™ p-nitrophenyl phosphate disodium salt (PNPP; Thermo Scientific, MA, USA) solution was added. The plate was incubated for 30 min at RT, and then the absorbance was measured at 450 nm.

### Reticulocyte enrichment

Reticulocytes were enriched from umbilical cord blood using a 19% Nycodenz solution (Axis-Shield, Oslo, Norway) in high-KCl buffer with gradient centrifugation as mentioned previously [29]. Briefly, fresh cord blood was washed twice with incomplete Roswell Park Memorial Institute (RPMI) 1640 medium, and white blood cells were removed by a non-woven fabric (NWF filter (ZhiXing Bio S&T Co. Ltd., Bengbu, China). After centrifugation, the packed cells were resuspended in high-KCl buffer (115 mM KCl, pH 7.4) and then incubated at 4 °C for 3 h with rotation. Each of 5 ml of the red blood cell (RBC)-high-KCl buffer mixture was overlaid on Nycodenz solution (19%, 3 ml) in a 15-ml tube. After centrifugation at 3000×*g* for 30 min without braking, the enriched reticulocytes in the interface layer were collected and washed three times with incomplete RPMI 1640 medium. The purity of reticulocytes was evaluated using thin blood smears with new methylene blue stain using light microscopy and thiazole orange (TO) (Becton Dikinson, San Jose, CA, USA) staining with flow cytometric analysis. A total of 100,000 events were obtained from each sample using a fluorescence-activated cell sorting (FACS) Accuri™ C6 Flow Cytometer (Becton Dikinson, Mansfield, MA, USA).

### Flow cytometric analysis of CD36 expression on erythrocytes

Samples for flow cytometric experiments were prepared as described previously with slight modifications [24]. The enriched reticulocytes were washed three times with PBS containing 1% bovine serum albumin (BSA), and then 1 million RBCs were incubated with 5 μg of PE/Cyanine7 anti-human CD36 (BioLegend, San Diego, CA, USA) or the same amount of PE/Cyanine7 Mouse IgG2 α,κ isotype control antibody as a control (BioLegend) for 30 min at 4 °C in the dark. After washing three times with PBS-1% BSA, the cells were incubated with TO for 30 min at RT. A total of 100,000 events were obtained per sample using a FACS Accuri™ C6 Flow Cytometer.

### Flow cytometry-based erythrocyte binding assay

The reticulocyte binding assay was performed as mentioned previously [30]. Briefly, 1 × 10^6^ cells were incubated with a gradient concentration of purified His-tagged recombinant PvMTRAP for 3 h at 25 °C. The PvRBP2b-His and glutathione S-transferase (GST)-His proteins were used in parallel as positive and negative controls, respectively. After incubation, the samples were washed with 200 µl of PBS-1% BSA twice and then incubated with 5 µg of APC anti-His Tag monoclonal antibody (BioLegend, San Diego, CA, USA) in 100 µl staining volume for 1 h at 4 °C in the dark. The samples were washed three times with PBS-1% BSA and incubated with TO for 30 min at 25 °C. A total of 100,000 events were counted per sample using a FACS Accuri™ C6 Flow Cytometer. All flow cytometric data were analyzed by FlowJo software (TreeStar, Ashland, OR, USA).

### Octet^®^ bio-layer interferometry analysis

The interactions between His-tagged *P. vivax* ligands and Fc-tagged reticulocyte receptors were assessed using protein G (ProG) biosensors in the Octet RED96 system (ForteBio Inc., Menlo Park, CA, USA). The Fc-tagged reticulocyte receptors were immobilized on the ProG biosensor, and then a twofold serial dilution of parasite ligands in HBS was loaded into the biosensor to generate the kinetic parameters. The data were analyzed with Octet System Data Analysis Software ver. 7.0. (Sartorius, Göttingen, Germany).

### Statistical analysis

The data were calculated using GraphPad Prism (GraphPad Software, San Diego, CA, USA) and Microsoft Excel 2016 (Microsoft, Redmond, WA, USA). For comparison, paired Student’s *t*-tests were utilized, and a *P*-value of < 0.05 was considered to indicate significance.

## Results

### PvMTRAP interacts specifically with CD36

**Fig. 1:**
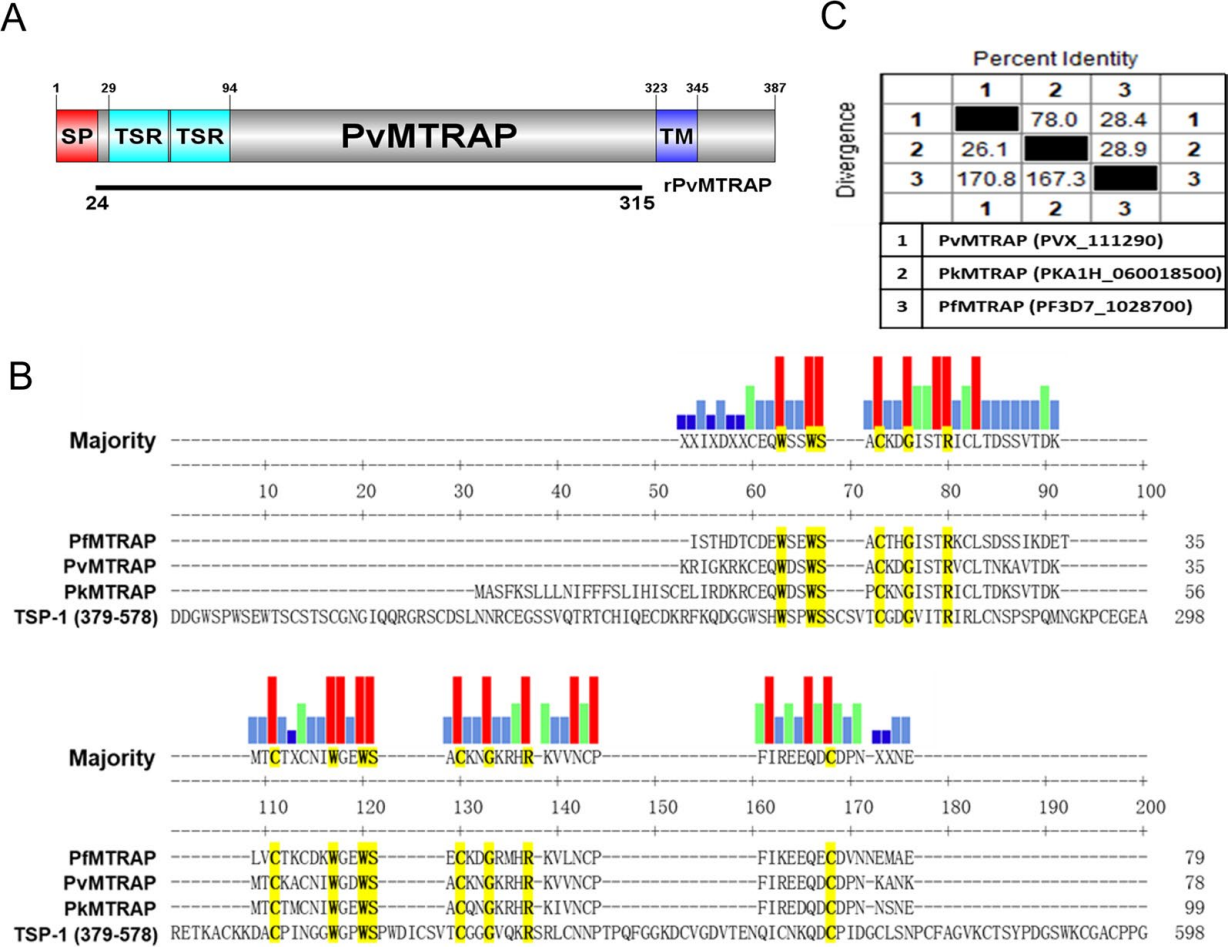
Schematic structure and sequence alignment of PvMTRAP. **A** Schematic structure of PvMTRAP. The full-length ectodomain of PvMTRAP was expressed with a HEK mammalian cell system. *SP* signal peptide, *TSR* thrombospondin repeat, *TM* transmembrane. **B** Clustal alignment of the TSR domains of MTRAP proteins from *P. vivax* (Pv), *P. knowlesi* (Pk), *P. falciparum* (Pf), and TSP-1 human protein. *Red*, *green*, *sky-blue*, and *dark-blue* bars indicate the conserved amino acids (aa) in four, three, two, and one sequences, respectively. The conserved aa are highlighted in yellow. **C** Percent identity and divergence (in percent) of the full-length ectodomains of PvMTRAP, PkMTRAP, and PfMTRAP

**Fig. 2:**
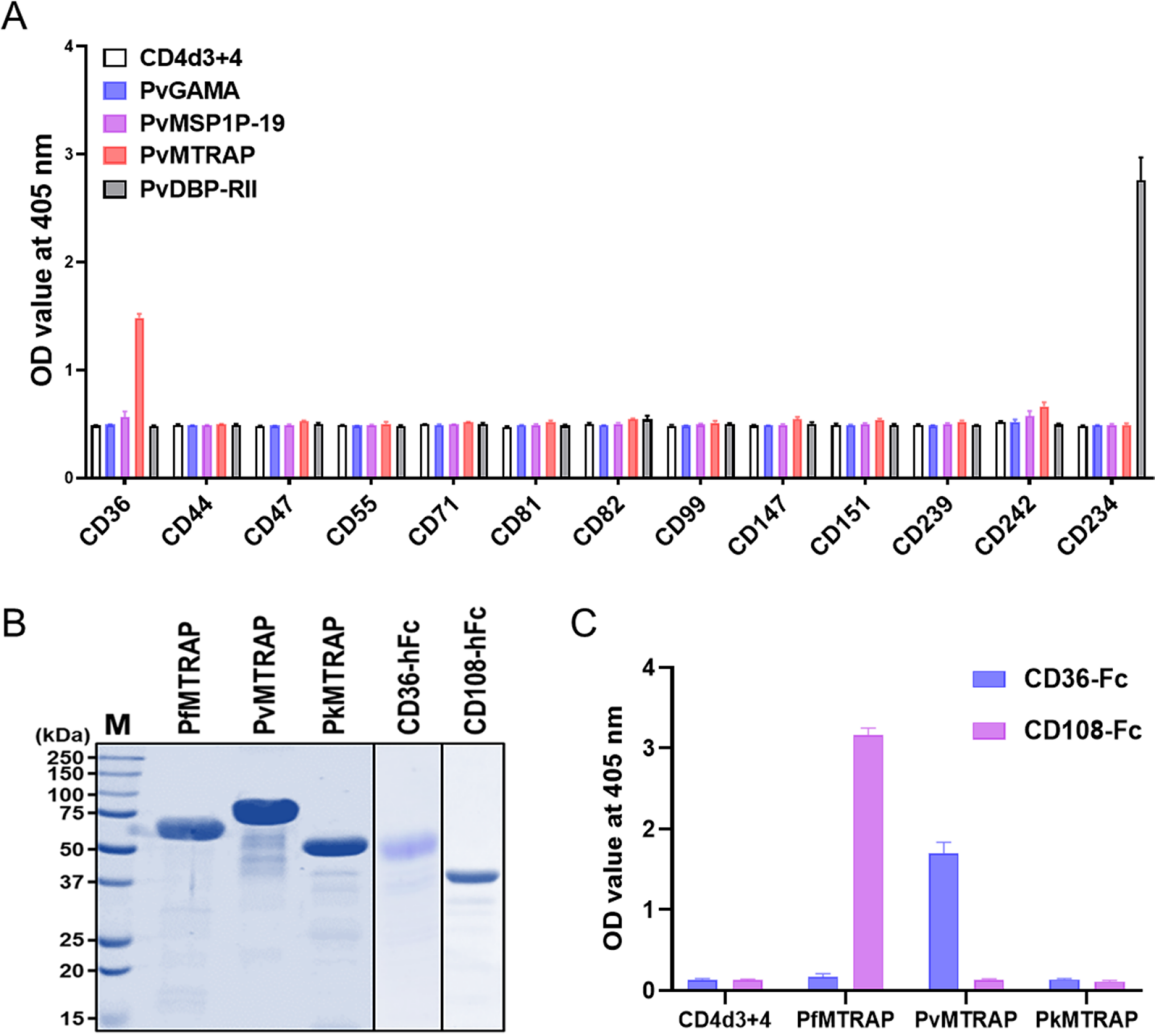
Binding specificity of PvMTRAP to CD36. **A** Systematic interaction screening between major functional *P. vivax* ligands and reticulocyte-abundant receptor proteins. PvDBP-RII binds to CD234 (human DARC) and was used as a positive control. **B** SDS‒PAGE of purified MTRAP proteins and their proven/suggested erythrocyte receptors. **C** Ability of MTRAP from various human malaria parasites to bind to CD36 as evaluated by ELISA**.** PfMTRAP and CD108 were utilized as positive controls. CD4d3 + 4 was used as a negative control for all experiments

PvMTRAP was predicted to be a transmembrane type 1 protein with a signal peptide at the N-terminus (Fig. 1A). A repeated sequence of ‘WxxWSxCxxGxxxR ~ C’ (where ‘**x**’ can be replaced by any amino acid) was conserved among MTRAP proteins from various human-infected malaria parasites and the TSR domain of the TSP-1 human protein (Fig. 1B). PvMTRAP and *Plasmodium knowlesi* MTRAP shared 78.0% sequence identity; however, neither was closely phylogenetically related to *P. falciparum* MTRAP (Fig. 1C). Avidity-based extracellular interaction screening (AVEXIS) method had been successfully applied to unravel novel ligand–receptor included in the erythrocyte invasion by *Plasmodium* spp. merozoite [31, 32]. In the present study, we applied the AVEXIS method with dimer Fc-tagged proteins functioning as prey proteins. Proteins that expressed at a higher level on immature erythrocytes than on normocytes were reviewed from previous studies, and 18 candidates were selected for recombinant protein expression [13, 25, 33]. Since the attempt to express CD35, CD49d, CD236R, CD238, and CD235a was unsuccessful, 13 reticulocyte abundant proteins were expressed in Fc-fusion form [13, 25, 31–35] (Additional file 1: Fig. S1A, Additional file 5: Table S1) and allowed to react with immobilized bait proteins PvMTRAP, PvGAMA, or PvMSP1P-19. Aside from PvDBP and CD234 (Duffy antigen receptor for chemokines, DARC) as the positive controls, only PvMTRAP and CD36 showed an interaction (Fig. 2A). Recombinant proteins were incorporated with rat CD4d3 + 4 domains, which likely increased the solubility of the proteins and enabled them to be detected with a mouse anti-rat CD4 monoclonal antibody (clone: OX68) for normalization purposes. Hence, a recombinant protein with only CD4d3 + 4 domains and appropriate tags was used as a negative control. The attempt to perform the AVEXIS assay in the reverse orientation of bait and prey proteins was unsuccessful since the CD36 could not be expressed as a bait protein by our system. CD36 was capable of binding to the human TSP-1 protein via the TSR domain [26], which exists in MTRAP proteins of *Plasmodium* spp. (Fig. 1B). To analyze the specific molecular interaction of PvMTRAP and CD36, we next included MTRAP proteins from different human-infecting malaria in the same ELISA methodology. Recombinant MTRAP proteins were expressed and purified (Fig. 2B) for this ELISA, and the binding of PfMTRAP to CD108 (semaphorin 7A) was used as a positive control. Interestingly, only PvMTRAP, not PkMTRAP or PfMTRAP, specifically interacted with CD36 (Fig. 2C). These binding differences might result from structural and electrostatic differences derived from the regions consisting of dense negatively charged amino acids (glutamic acid) of PfMTRAP and several amino acids substitution of PkMTRAP sequence compared to PvMTRAP (Additional file 2: Fig. S2). Whereas the TSR domain from PvMTRAP and PkMTRAP shared 81.8% sequence identity, sequence alignment using three distinct strains of *P. vivax* and *P. knowlesi* indicated several conserved amino acids substitution that could be accounted for the difference in the binding to CD36 (Additional file 3: Fig. S3A–B). However, it may need additional confirmation in further study.

### CD36 is selectively abundant on the reticulocyte surface, as confirmed by flow cytometry

Previous studies using either proteomic or flow cytometry-based assays have suggested that CD36 is exclusively expressed in immature erythrocytes than in mature erythrocytes [13, 24, 25, 36]. Moreover, CD36 was inconsistently present on reticulocytes, since younger reticulocytes (high CD71 level) expressed higher levels of CD36 [25]. Therefore, determining CD36 expression of enriched reticulocyte samples used for downstream erythrocyte binding assay would contribute valuably to assessing the role of CD36 as a reticulocyte receptor.

**Fig. 3:**
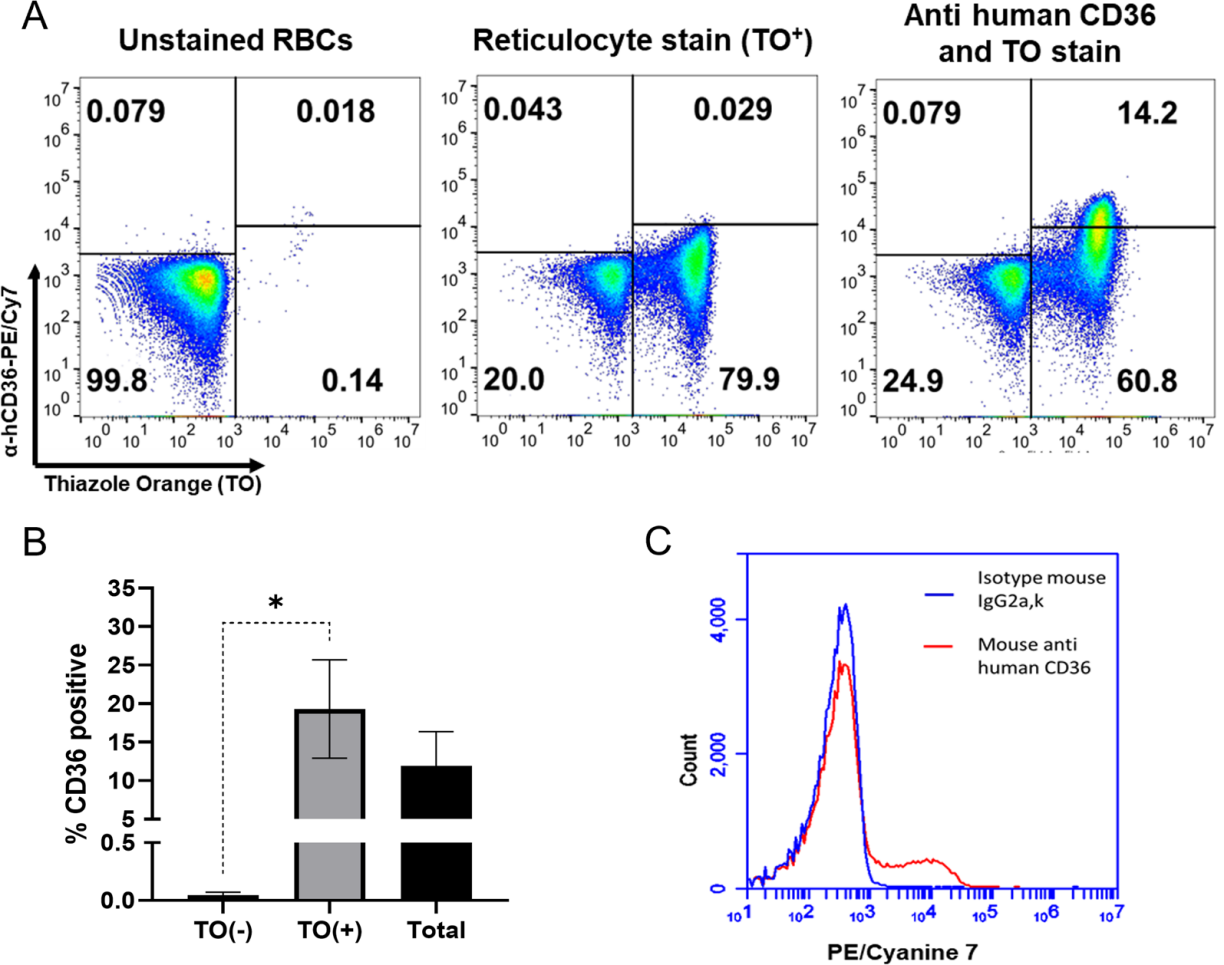
CD36 is selectively abundant on the surfaces of reticulocytes. **A** Dot plot diagrams showing the unstained RBCs (left) and RBCs single stained with thiazole orange (TO) (center) for gating. The application of this gating strategy to RBC dual staining with TO and anti-human CD36 antibody is indicated in the right diagram. Numbers in quadrants represent gate frequencies. **B** CD36 was detected on 19.34 ± 2.14% of reticulocytes [TO (+)] but mostly disappeared during the maturation of RBCs (0.30 ± 0.09% of normocytes [TO (−)] were positive for CD36). Among the reticulocyte-enriched RBCs used for the downstream binding assay (total), 11.35 ± 3.23% of cells expressed CD36. The bars represent the mean ± SEM from three biological replicates. Single asterisks, *P* < 0.05. **C** Histogram of the fluorescence signal from a single stain with a mouse anti-human CD36 antibody (red) and its isotype control (blue)

Here, we enriched reticulocytes from cord blood and then performed flow cytometry to analyze CD36 expression on the erythrocyte surface (Fig. 3A). There was 19.34 ± 2.14% of reticulocytes expressed CD36; however, this extensively decreased when the erythrocytes matured (0.30 ± 0.09% of normocytes were positive for CD36) (mean ± standard error of the mean [SEM] in triplicate biological repeats) (Fig. 3B). The isotype of mouse anti-human CD36 antibody was included under the same conditions and showed no positive signal (Fig. 3C, blue histogram), indicating that the anti-human CD36 antibody bound specifically to its target antigen on erythrocytes (Fig. 3C, red histogram).

### PvMTRAP preferentially binds to immature erythrocytes

**Fig. 4:**
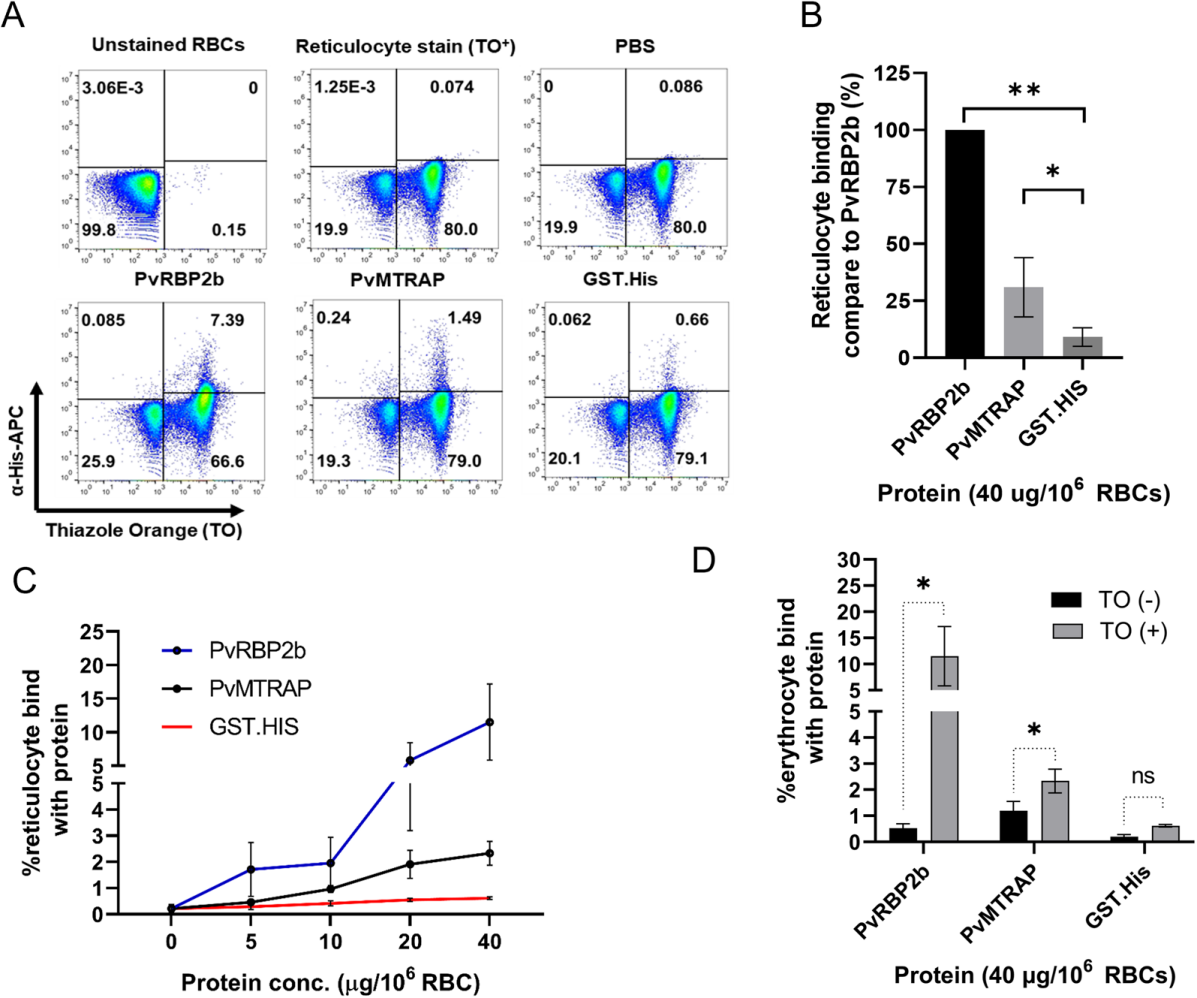
Binding activity of PvMTRAP to reticulocytes. **A** Gating strategy based on unstained RBCs (upper left) and RBCs stained with TO (upper center). RBCs (1 × 10^6^) were incubated with PBS (upper right) or 40 μg of PvRBP2b (lower left), PvMTRAP (lower center), or GST-His (lower right) before being co-stained with an anti-His-APC antibody and TO. **B** The reticulocyte binding ability of PvMTRAP was higher than that of GST-His (30.98 ± 12.99% and 9.08 ± 4.05% relative to PvRBP2b, respectively). **C** Serial dilutions of PvRBP2b (blue), PvMTRAP (black), or GST-His (red) proteins were examined for reticulocyte binding. **D** PvMTRAP preferentially binds to reticulocytes over normocytes. The data are shown as the mean ± SEM from three biological replicates in twice technical repeats. Single asterisks, *P* < 0.05; double asterisks, *P* < 0.01

While PvMTRAP interacts specifically with CD36, which selectively exists on immature erythrocytes, this parasite ligand is predicted to bind to red blood cells with a preference for reticulocytes. We prepared erythrocyte samples with 57.66 ± 12.23% reticulocytes and 11.35 ± 3.23% cells expressing CD36 for a downstream flow cytometry-based erythrocyte binding assay (Fig. 3C). PvRBP2b (residues 169–813) binds specifically to a well-known reticulocyte marker CD71 [14] and was utilized as a positive control, and recombinant GST-His-tagged protein was used as a negative control (Additional file 4: Fig. S4A–B). The reticulocyte binding ability of PvMTRAP was 30.98 ± 12.99 as a percentage relative to that of PvRBP2b, which was significantly higher than that of GST-His-tagged protein (9.08 ± 4.05%, *P* = 0.018) (Fig. 4A–B). PvMTRAP was capable of binding to reticulocytes in a concentration-dependent manner (Fig. 4C). In accordance with the PvRBP2b results, PvMTRAP preferentially bound to reticulocytes rather than normocytes (Fig. 4D).

### Quantitative evaluation of the interaction between PvMTRAP and CD36

**Fig. 5:**
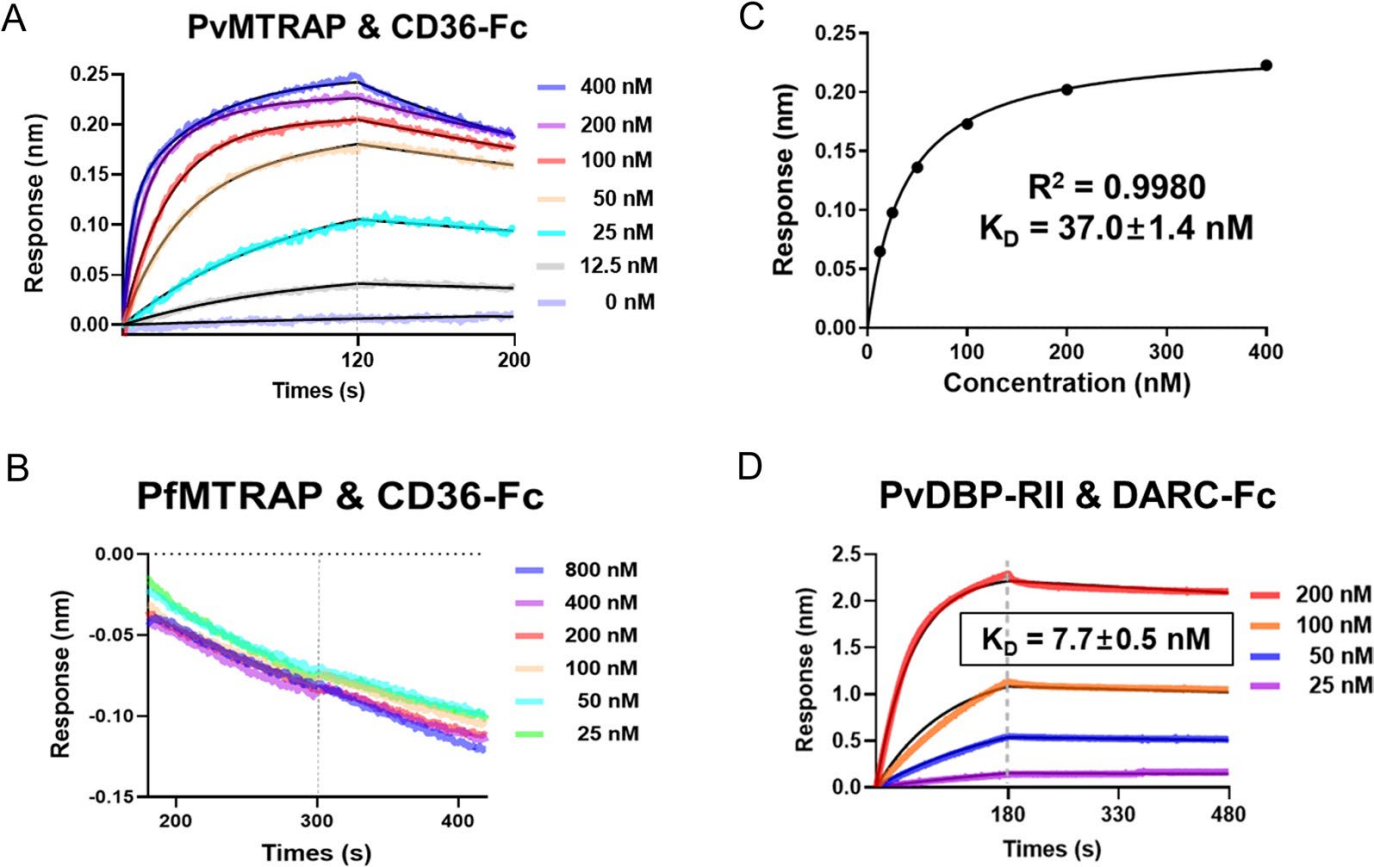
Quantitative analysis of the interactions of MTRAP proteins with CD36 with a protein‒protein interaction analyzer. Serial dilutions of PvMTRAP (**A**) or PfMTRAP (**B**) were allowed to react with immobilized CD36-Fc on a chip, and only PvMTRAP and CD36-Fc formed a binding pattern. **C** The response in equilibrium was plotted against the related PvMTRAP concentration, and a *K*_D_ of 37.0 ± 1.4 nM was calculated (coefficient of determination [*r*^2^] = 0.998). **D** PvDBP-RII and DARC were utilized as positive controls. The *K*_D_ was presented as mean and SEM from three replicates

To confirm the direct binding of PvMTRAP to CD36 and to quantify this interaction, we used BLI (the Octet system). Twofold serial dilutions of the MTRAP protein were allowed to interact with CD36-Fc immobilized on the ProG biosensor, and the results showed that PvMTRAP specifically generated concentration-dependent binding to CD36 (Fig. 5A–B). The response in equilibrium was plotted against the related PvMTRAP concentration, and a *K*_D_ of 37.0 ± 1.4 nM was calculated (coefficient of determination [*r*^2^] = 0.998) (Fig. 5C). The interaction between CD234 (human DARC) and PvDBP-RII (*K*_D_ = 7.7 ± 0.5 nM) was used as a positive control (Fig. 5D) (Additional file 1: Fig. S1B).

## Discussion

The interactions between malaria parasites and human CD36 have been well documented, especially in the pathogenesis of *P. falciparum* infection [23, 37]. However, previous studies have only addressed the CD36 expressed on endothelial cells to which parasitized erythrocytes bind, which triggers downstream cyto-adhering-related pathobiological processes [23, 38]. CD36 is expressed in various human cell types, including erythrocytes, and it is supposed that this receptor is selectively present on reticulocytes, which are host cells of *P. vivax*. MTRAP contains a conserved TSR domain, which is a well-known ligand of CD36, supporting the hypothesis that PvMTRAP may interact with human CD36 as a ligand receptor for the invasion of *P. vivax*. Hence, we included these proteins in our systematic screening methodology, and the results indicated that CD36 interacted with PvMTRAP. Furthermore, this interaction was specific at the molecular level in in vitro analysis, as MTRAP proteins from other human-infecting malaria parasites were unable to bind to CD36. To quantify the strength of the interaction, a BLI system was utilized, and we determined that PvMTRAP binds to CD36 with a *K*_D_ of 37.0 ± 1.4 nM, which is comparable to that of the PvDBP and human DARC interaction (K_D_: 7.7 ± 0.5 nM). MTRAP proteins from other human malaria parasites were also examined in this BLI experiment and did not bind to CD36, addressing any concern about the weakness of ELISA in detecting weak interactions. Our findings were in line with a previous report about PfMTRAP and semaphorin-7A, suggesting that although MTRAP proteins from different human-infected malaria parasites share conserved TSR sequences and may utilize the TSR domain in binding to their erythrocyte receptors, this kind of interaction could be heterogeneous among *Plasmodium* spp. [22]. Altogether, the biomolecular interaction data provided robust evidence for the specific binding of PvMTRAP to human CD36.

One of the barriers to using recombinant proteins as research models is the inability of these proteins to completely recapitulate native proteins. Recombinant proteins may be insufficiently folded and lack some structural and functional characteristics of native proteins, especially those related to interactions with other molecules [39, 40]. To minimize this limitation, we used a mammalian cell-based protein expression model based on its ability to induce the highest level of post-translational processing and functional activity of protein [40]. Moreover, we further successfully established a cellular model for evaluating CD36 interactions using human erythrocytes. Reticulocytes were enriched from umbilical cord blood and were first included in a flow cytometry-based assay to assess CD36 expression on their surface. As expected, nearly one-fifth of reticulocytes expressed CD36, while normocytes that presented this receptor were rare (Fig. 3B). Our report contributes additional proof-of-concept data illustrating that CD36 is a reticulocyte marker, while this receptor is selectively abundant on reticulocytes and progressively disappears as reticulocytes mature into normocytes. The reasonable expression level of native CD36 on reticulocytes makes these cells an appropriate cellular model for assessing the protein‒protein interactions of CD36. Other cellular systems enable CD36 to be analyzed on their surface, such as THP-1 cell systems; however, they usually require additional procedures to enhance the expression of CD36 [41]. In addition, reticulocytes might be preferred, as CD36 was investigated concordantly with ligands from the malaria parasite, and the result can be interpreted directly with regard to malaria pathophysiology. Applying this approach, we found that recombinant PvMTRAP was capable of binding to reticulocytes at nearly one-third of the level of PvRBP2b (Fig. 4B). The binding of PvMTRAP to reticulocytes was significantly higher than that to normocytes, which was in accordance with the known properties of CD36 on the surfaces of erythrocytes, supporting the biochemical characteristics of the interaction between PvMTRAP and CD36. Our unpublished data revealed that PvMTRAP may be involved in erythrocyte invasion, since antibodies against PvMTRAP exhibited activity in invasion inhibition assay. Combined with the exclusive expression of CD36 on reticulocytes, the newly described interaction between PvMTRAP and CD36 warrants further study for intensive characterization as a ligand and receptor for reticulocyte invasion by *P. vivax*.

## Conclusions

In summary, PvMTRAP specifically interacts with CD36 with a comparable binding affinity to that between PvDBP and human DARC. Furthermore, by binding preferentially to reticulocytes on which CD36 is selectively present, PvMTRAP may serve as a ligand of the reticulocyte receptor CD36 for the invasion of *P. vivax*.

### Supplementary Information

**Additional file 1: Figure S1**. (**A**) SDS‒PAGE analysis of 13 recombinant proteins that are abundant on reticulocytes and were expressed and purified based on the Fc-tag. Different migration of protein under reducing (R) and non-reducing (N) conditions confirmed the existence of the Fc-tag. (**B**) Quality assessment of PvDBP-RII using ELISA and BLI experiments.

**Additional file 2: Figure S2**. The three *Plasmodium* species of MTRAP protein sequences were aligned. There were two repeated sequence patterns (black square), and especially, there were condensed negative charged amino acids regions in PfMTRAP different from other *Plasmodium* species (red square).

**Additional file 3: Figure S3**. (**A**) Percent identity of TSR domain from PvMTRAP and PkMTRAP. (**B**) The TSR domains of MTRAP protein from three distinct strains of *P. vivax* and *P. knowlesi* were aligned. The disagreement between the TSR domain of PvMTRAP and PkMTRAP was conserved.

**Additional file 4: Figure S4**. SDS‒PAGE and western blot analysis of the purified recombinant proteins used for the reticulocyte binding assay. (**A**) The quality of purified His-tagged recombinant PvRBP2b_**169–813**_ (Lane 1), GST (Lane 2), and PvMTRAP (Lane 3) was assessed by SDS‒PAGE. (**B**) Western blot assay using an anti-penta-His antibody to capture PvRBP2b (Lane 1), GST (Lane 2), and PvMTRAP (Lane 3).

**Additional file 5: Table S1.** The human reticulocyte ectodomain protein library. Exception for CD108 as a positive control, entire ectodomain from remaining proteins were expressed. A Fc-tag (around 25 kDa) was incorporated into proteins.

## Data Availability

The data supporting the findings of the study must be available within the article and/or its supplementary materials, or deposited in a publicly available database.

## Abbreviations

SDS‒PAGE: Sodium dodecyl sulfate–polyacrylamide gel electrophoresis
MTRAP: Merozoite-specific thrombospondin-related anonymous protein
CD36: Cluster of differentiation 36
PBS-T: Phosphate-buffered saline with Tween 20
TO: Thiazole orange
BSA: Bovine serum albumin
