# Supplementary figures and images for: Plasmodium vivax merozoite-specific thrombospondin-related anonymous protein (PvMTRAP) interacts with human CD36, suggesting a novel ligand–receptor interaction for reticulocyte invasion
Source: Parasit Vectors. 2023 Nov 19;16:426. doi: 10.1186/s13071-023-06031-5 (PMC10658926; doi:10.1186/s13071-023-06031-5)

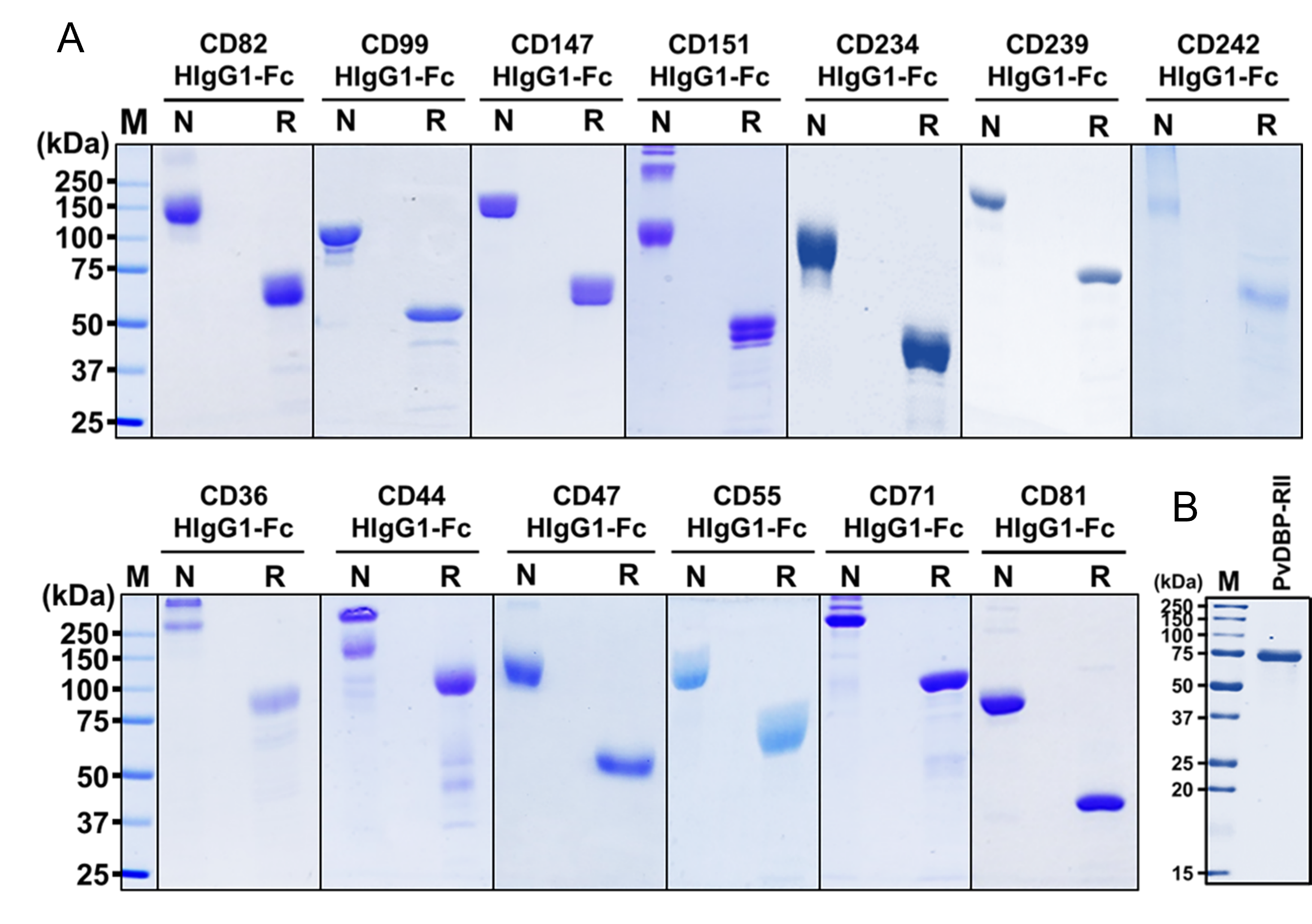

Supplement: Supplementary file 1 — Additional file 1: Figure S1. (A) SDS‒PAGE analysis of 13 recombinant proteins that are abundant on reticulocytes and were expressed and purified based on the Fc-tag. Different migration of protein under reducing (R) and non-reducing (N) conditions confirmed the existence of the Fc-tag. (B) Quality assessment of PvDBP-RII using ELISA and BLI experiments. [file 13071_2023_6031_MOESM1_ESM.tif]

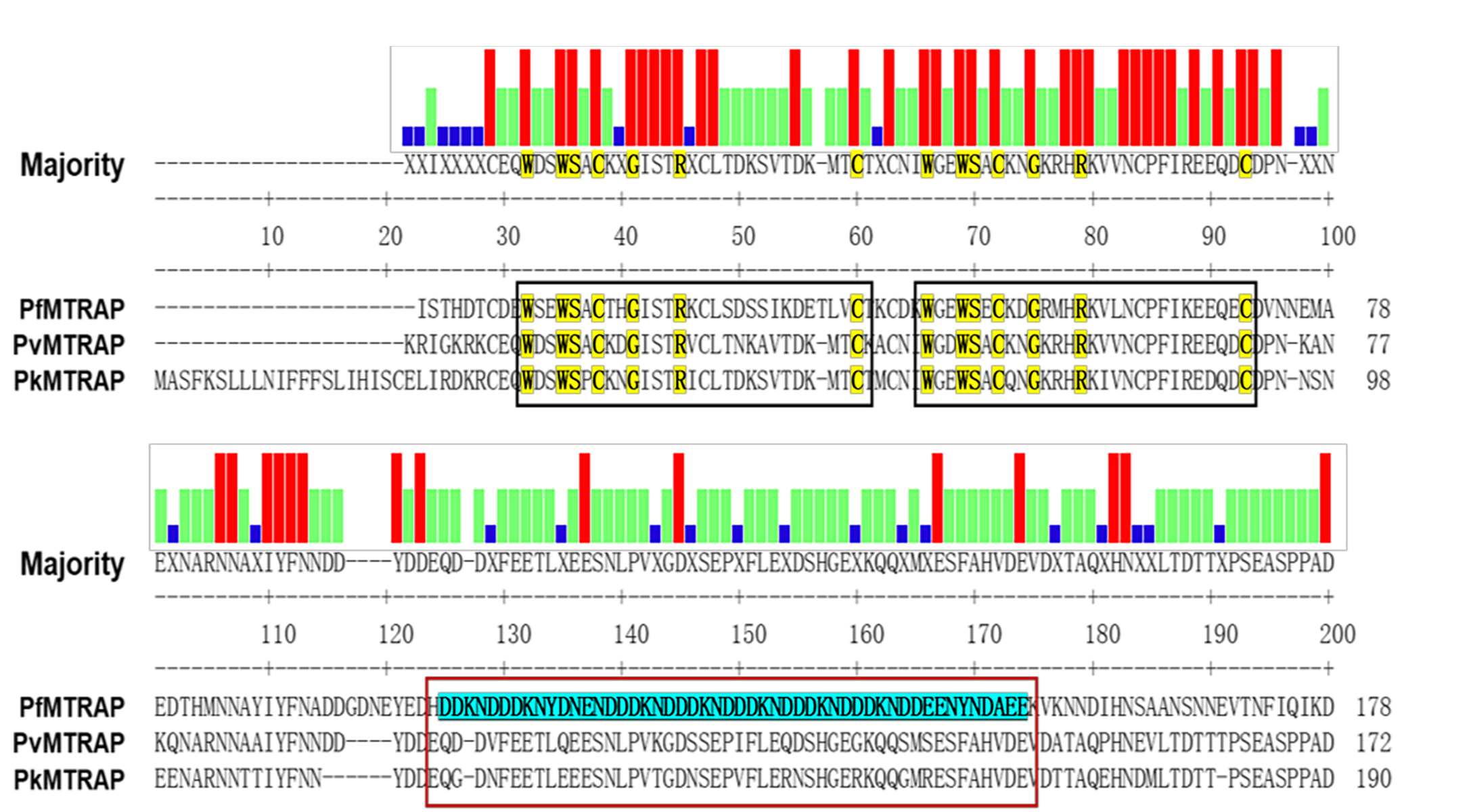

Supplement: Supplementary file 2 — Additional file 2: Figure S2. The three Plasmodium species of MTRAP protein sequences were aligned. There were two repeated sequence patterns (black square), and especially, there were condensed negative charged amino acids regions in PfMTRAP different from other Plasmodium species (red square). [file 13071_2023_6031_MOESM2_ESM.tif]

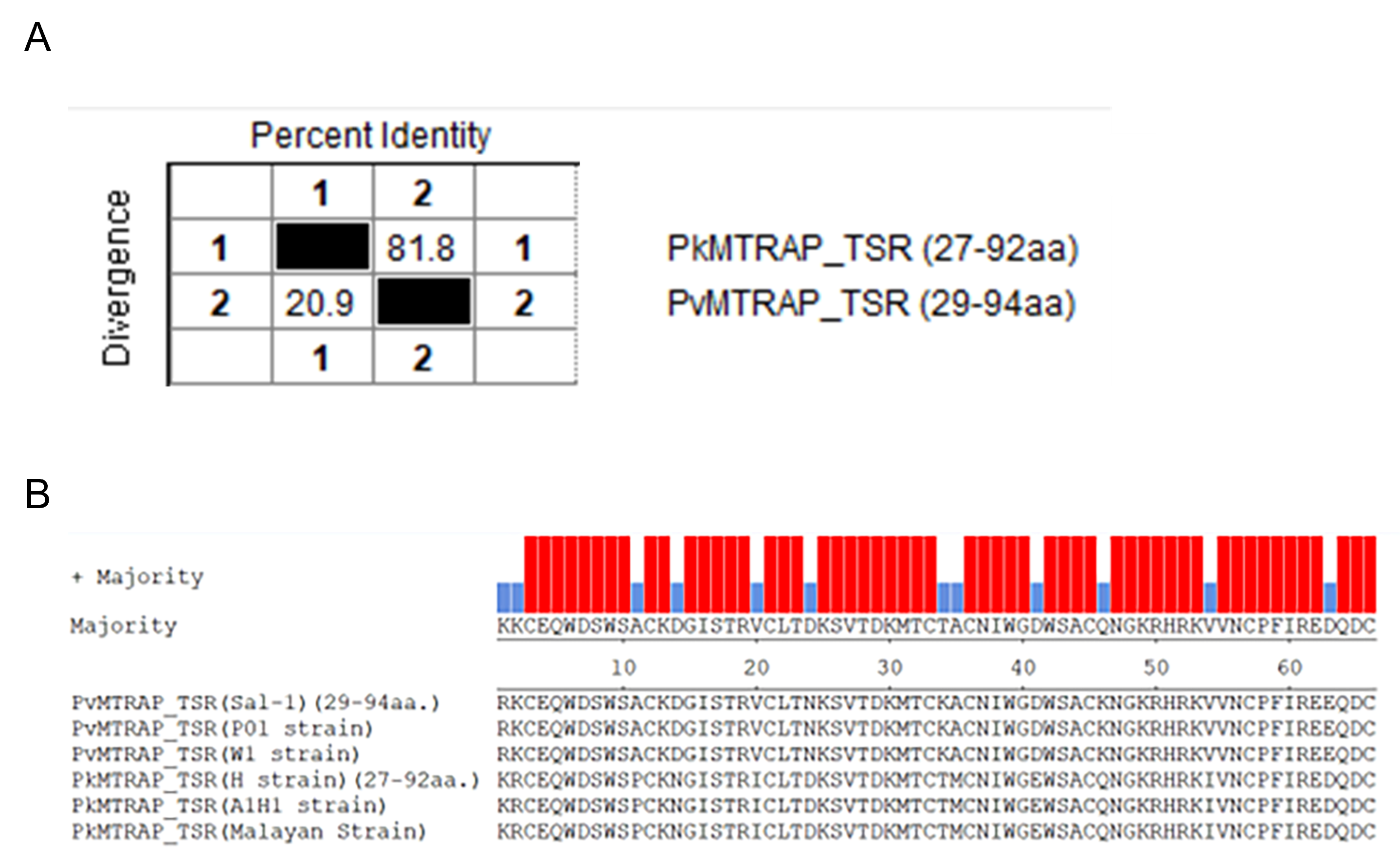

Supplement: Supplementary file 3 — Additional file 3: Figure S3. (A) Percent identity of TSR domain from PvMTRAP and PkMTRAP. (B) The TSR domains of MTRAP protein from three distinct strains of P. vivax and P. knowlesi were aligned. The disagreement between the TSR domain of PvMTRAP and PkMTRAP was conserved. [file 13071_2023_6031_MOESM3_ESM.tif]

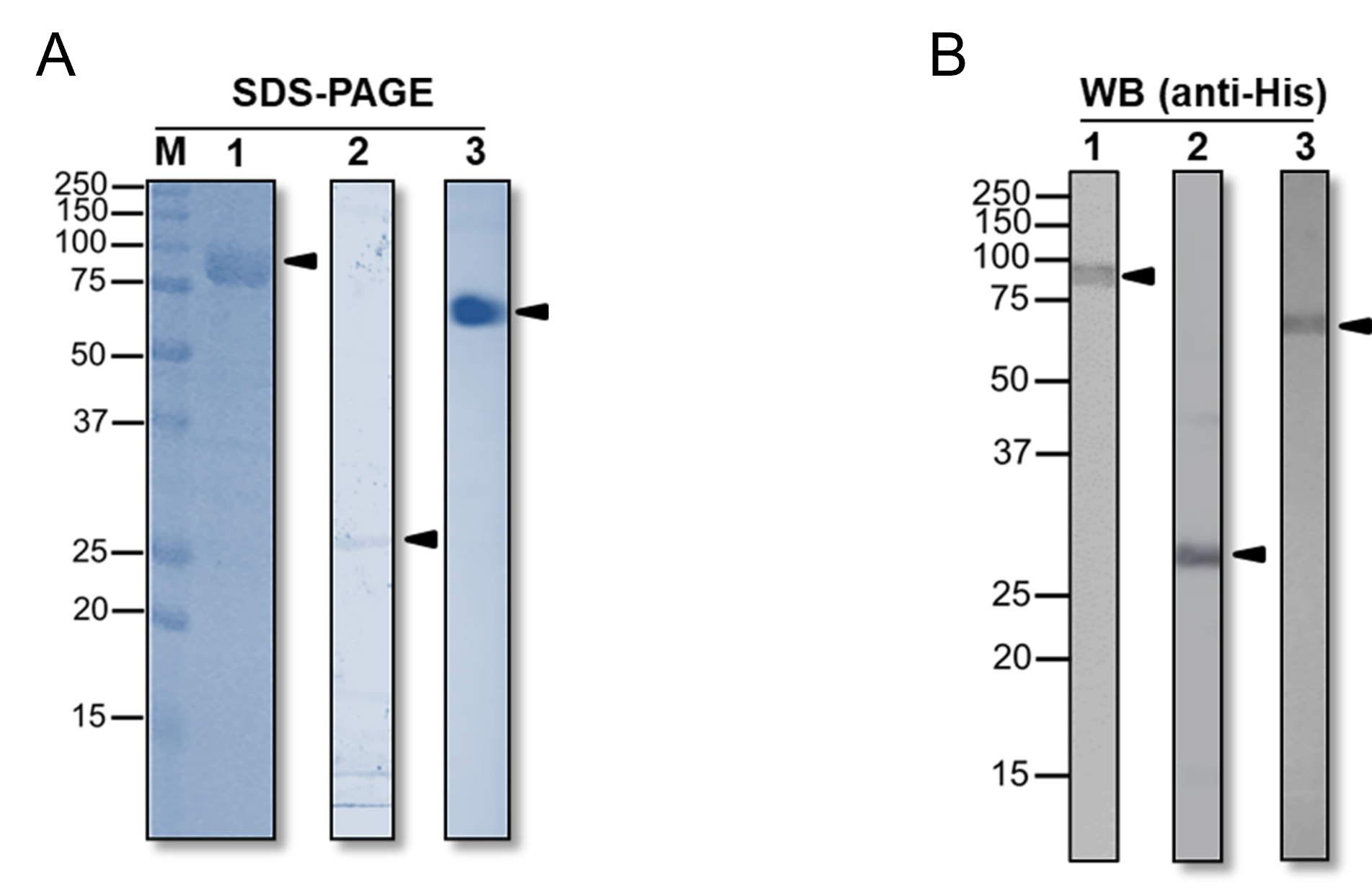

Supplement: Supplementary file 4 — Additional file 4: Figure S4. SDS‒PAGE and western blot analysis of the purified recombinant proteins used for the reticulocyte binding assay. (A) The quality of purified His-tagged recombinant PvRBP2b169–813 (Lane 1), GST (Lane 2), and PvMTRAP (Lane 3) was assessed by SDS‒PAGE. (B) Western blot assay using an anti-penta-His antibody to capture PvRBP2b (Lane 1), GST (Lane 2), and PvMTRAP (Lane 3). [file 13071_2023_6031_MOESM4_ESM.tif]
