# Supplementary material for: Plasmodium vivax merozoite-specific thrombospondin-related anonymous protein (PvMTRAP) interacts with human CD36, suggesting a novel ligand–receptor interaction for reticulocyte invasion
Source: Parasit Vectors. 2023 Nov 19;16:426. doi: 10.1186/s13071-023-06031-5 (PMC10658926; doi:10.1186/s13071-023-06031-5)
Supplement: Supplementary file 5 — Additional file 5: Table S1. The human reticulocyte ectodomain protein library. Exception for CD108 as a positive control, entire ectodomain from remaining proteins were expressed. A Fc-tag (around 25 kDa) was incorporated into proteins. [file 13071_2023_6031_MOESM5_ESM.docx]

**Additional file Table 1**

| **Sample number** | **Name** | **GenBank accession number** | **Expression regions (aa.)** | **Predicted size w/o tag (kDa)** |
| --- | --- | --- | --- | --- |
| 1 | CD36 | P16671 | 29-439 | 48.6 |
| 2 | CD44 | P16070 | 1-267 | 29.2 |
| 3 | CD47 | Q08722 | 1-139 | 15.4 |
| 4 | CD55 | P08174 | 1-353 | 38.5 |
| 5 | CD71 | P02786 | 8-679 | 75.2 |
| 6 | CD81 | BC002978 | 1-236 | 25.8 |
| 7 | CD82 | P27701 | 1-267 | 29.6 |
| 8 | CD99 | P14209 | 1-124 | 12.4 |
| 9 | CD108 | O75326 | 1-644 | 72.6 |
| 10 | CD147 | P35613_1 | 1-206 | 22.2 |
| 11 | CD151 | P48509 | 1-253 | 28.3 |
| 12 | CD234 | Q16570 | 1-63 | 38 |
| 13 | CD239 | P50895 | 1-548 | 59.4 |
| 14 | CD242 | Q14773 | 1-271 | 29.3 |
| 15 | PvMTRAP | PVX_111290 | 24-315 | 32.4 |
| 16 | PkMTRAP | PKNH_0613400 | 23-310 | 33.1 |
| 17 | PfMTRAP | PF3D7_1028700 | 23-432 | 47.8 |
| 18 | PvGAMA | PVX_088910 | 408-589 | 79.6 |
| 19 | PvMSP1P-19 | PVX_099975 | 1751-1834 | 9.6 |
| 20 | PvDBP-RII | PVX_110810 | 194-521 | 38.8 |
| 21 | CD4d3+4 | P05540 | 210-360 | 20.5 |
